# A Human Lung Xenograft Mouse Model of Nipah Virus Infection

**DOI:** 10.1371/journal.ppat.1004063

**Published:** 2014-04-03

**Authors:** Gustavo Valbuena, Hailey Halliday, Viktoriya Borisevich, Yenny Goez, Barry Rockx

**Affiliations:** 1 Department of Pathology, University of Texas Medical Branch, Galveston, Texas, United States of America; 2 Institute of Human Infections and Immunity, University of Texas Medical Branch, Galveston, Texas, United States of America; 3 Department Microbiology & Immunology, University of Texas Medical Branch, Galveston, Texas, United States of America; Johns Hopkins University, United States of America

## Abstract

Nipah virus (NiV) is a member of the genus *Henipavirus* (family *Paramyxoviridae*) that causes severe and often lethal respiratory illness and encephalitis in humans with high mortality rates (up to 92%). NiV can cause Acute Lung Injury (ALI) in humans, and human-to-human transmission has been observed in recent outbreaks of NiV. While the exact route of transmission to humans is not known, we have previously shown that NiV can efficiently infect human respiratory epithelial cells. The molecular mechanisms of NiV-associated ALI in the human respiratory tract are unknown. Thus, there is an urgent need for models of henipavirus infection of the human respiratory tract to study the pathogenesis and understand the host responses. Here, we describe a novel human lung xenograft model in mice to study the pathogenesis of NiV. Following transplantation, human fetal lung xenografts rapidly graft and develop mature structures of adult lungs including cartilage, vascular vessels, ciliated pseudostratified columnar epithelium, and primitive “air” spaces filled with mucus and lined by cuboidal to flat epithelium. Following infection, NiV grows to high titers (10^7^ TCID_50_/gram lung tissue) as early as 3 days post infection (pi). NiV targets both the endothelium as well as respiratory epithelium in the human lung tissues, and results in syncytia formation. NiV infection in the human lung results in the production of several cytokines and chemokines including IL-6, IP-10, eotaxin, G-CSF and GM-CSF on days 5 and 7 pi. In conclusion, this study demonstrates that NiV can replicate to high titers in a novel *in vivo* model of the human respiratory tract, resulting in a robust inflammatory response, which is known to be associated with ALI. This model will facilitate progress in the fundamental understanding of henipavirus pathogenesis and virus-host interactions; it will also provide biologically relevant models for other respiratory viruses.

## Author Summary

Nipah virus (NiV) is a highly pathogenic zoonotic virus that causes fatal disease in humans and a variety of other mammalian hosts including pigs. Given the lack of effective therapeutics and vaccines, this virus is considered a public health and agricultural concern, and listed as category C priority pathogen for biodefense research by the National Institute of Allergy and Infectious Diseases. Both animal-to-human and human-to-human transmission has been observed. Studies on the molecular mechanisms of NiV-mediated pathogenesis have been hampered by the lack of biologically relevant *in vivo* models for studying the initial host responses to NiV infection in the human lung. We show here a new small animal model in which we transplant human lung tissue for studying the pathogenesis of NiV. We showed that NiV can replicate to high levels in the human lung. NiV causes extensive damage to the lung tissue and induces important regulators of the inflammatory response. This study is the first to use a human lung transplant for studying infectious diseases, a powerful model for studying the pathogenesis of NiV infection, and will open up new possibilities for studying virus-host interactions.

## Introduction

Nipah virus (NiV) is a member of the genus *Henipavirus* (family *Paramyxoviridae*) that causes severe and often lethal respiratory illness and encephalitis in humans resulting in case fatality rates of up to 92% [1]. The first human cases of NiV infection were identified during an outbreak of severe febrile encephalitis in Malaysia and Singapore in 1998–1999 [2], [3]. More recently, outbreaks have occurred in Bangladesh and India almost yearly since 2001 [1], [4]. NiV can cause Acute Lung Injury (ALI) in humans, and human-to-human transmission has been observed in recent outbreaks of NiV [5], [6], [7]. Data on the histopathology of the lungs of NiV cases is limited to necropsy findings in the respiratory tract of NiV infected cases and include hemorrhage, necrosis and inflammation in the epithelium of the small airways but not in the bronchi [8].

Endothelial cells have been identified as a major target for NiV and most studies have focused on the role of the endothelium in NiV pathogenesis [9], [10], [11]. However very limited data is available on the host responses following NiV infection in the human lung. While the endothelium plays an important role in the terminal stages of NiV infection, the role of the respiratory epithelium in the early stages of infection is critical; however, it remains largely unexplored. The specific sites of henipavirus infection in the human respiratory tract are still unknown as well as the molecular mechanism by which these viruses cause disease in humans. We have previously shown that NiV can efficiently infect human respiratory epithelial cells from the trachea, bronchi and small airways resulting in the induction of key inflammatory mediators that have been implicated in leukocyte recruitment and ALI [12]. Similarly, in animal models (hamster, ferret and African green monkey), NiV can replicate to high titers in the lungs of these animals and cause acute and severe respiratory distress [13], [14], [15], [16], [17]. Human xenograft mouse models have previously been used to study tissue development and cancer as well as the pathogenesis of infectious agents [18], [19], [20]. The majority of viral pathogenesis studies involving human xenograft mice focus on Human Immunodeficiency Virus (HIV) or Human Cytomegalovirus (HCMV) in humanized mice that have been grafted with human hematopoietic stem cells and thymus [21], [22].Here we report the first characterization of NiV infection of the human respiratory tract using a human lung xenograft model to gain further insight into the mechanisms of NiV pathogenesis in humans. Our results showed that NiV replicates to high titers in the human lung and that infection results in the induction of a robust host response.

## Results

### Human lung xenograft development

**Figure 1:**
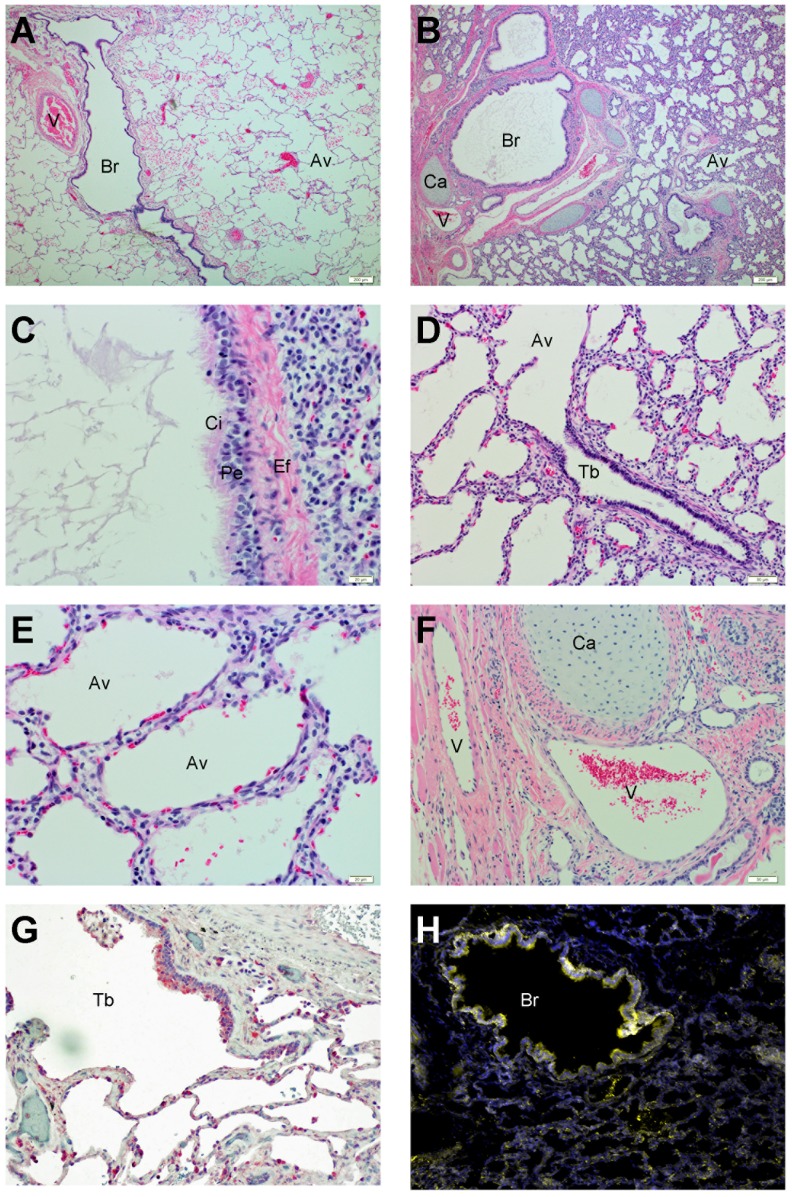
Histological characterization of human lung xenografts. Normal (A) and xenograft (B–F) human lung sections were stained with H&E as described in Experimental Procedures. Following transplantation, human lung xenografts develop mature structures (B) similar to those seen in normal human lung (A)(10× magnification). Structures include pseudostratified ciliated epithelium (C) (40× magnification), terminal bronchioles (D) (10× magnification), alveolar spaces (E)(40× magnification) and pulmonary vasculature (F)(10× magnification). Ephrin B2 expression was observed in normal human lung (red) (G) and human lung xenograft tissues (yellow) (H) with similar distribution on epithelium (10× magnification). Br = bronchi, V = pulmonary vasculature, Av = alveoli, Ca = cartilage, Ci = cilia, Pe = pseudostratified epithelium, Ef = elastic fibers, Tb = terminal bronchiole.

Severely immunodeficient NSG mice served as hosts to support the successful engraftment of human lung xenografts. We implanted 6 small fragments of human fetal lung in the dorsal subcutaneous space, 3 on each side of the spine. Following transplantation, human fetal lung xenografts typically increased in size by 2–10 fold over 3 months. The human fetal lung xenografts rapidly grafted and developed mature structures similar to those seen in adult lung (Figure 1A). These mature structures included bronchi that were partially surrounded by cartilage (Figure 1B), lined with ciliated pseudostratified columnar epithelium (Figure 1C) and surrounded by longitudinal elastic fibers. The bronchi divide into bronchioles and terminal bronchioles lined by cuboidal to flat epithelium (Figure 1D). The distal respiratory tract comprises of primitive alveolar spaces that are lined with both cells that have flat (type 1) and larger rounded (type 2) pneumocyte morphology (Figure 1E). The alveolar walls of xenografts were thicker than those of normal adult human lungs. The human graft was well vascularized with the presence of arteries, veins, and capillaries (Figure 1F). Finally, the expression patterns of ephrin B2, the receptor of NiV, was similar to that seen in normal human lung tissue [23] (Figure 1G) with expression on bronchial epithelium, alveolar cells and vasculatures (Figure 1H).

### NiV replication in human lung xenografts

**Figure 2:**
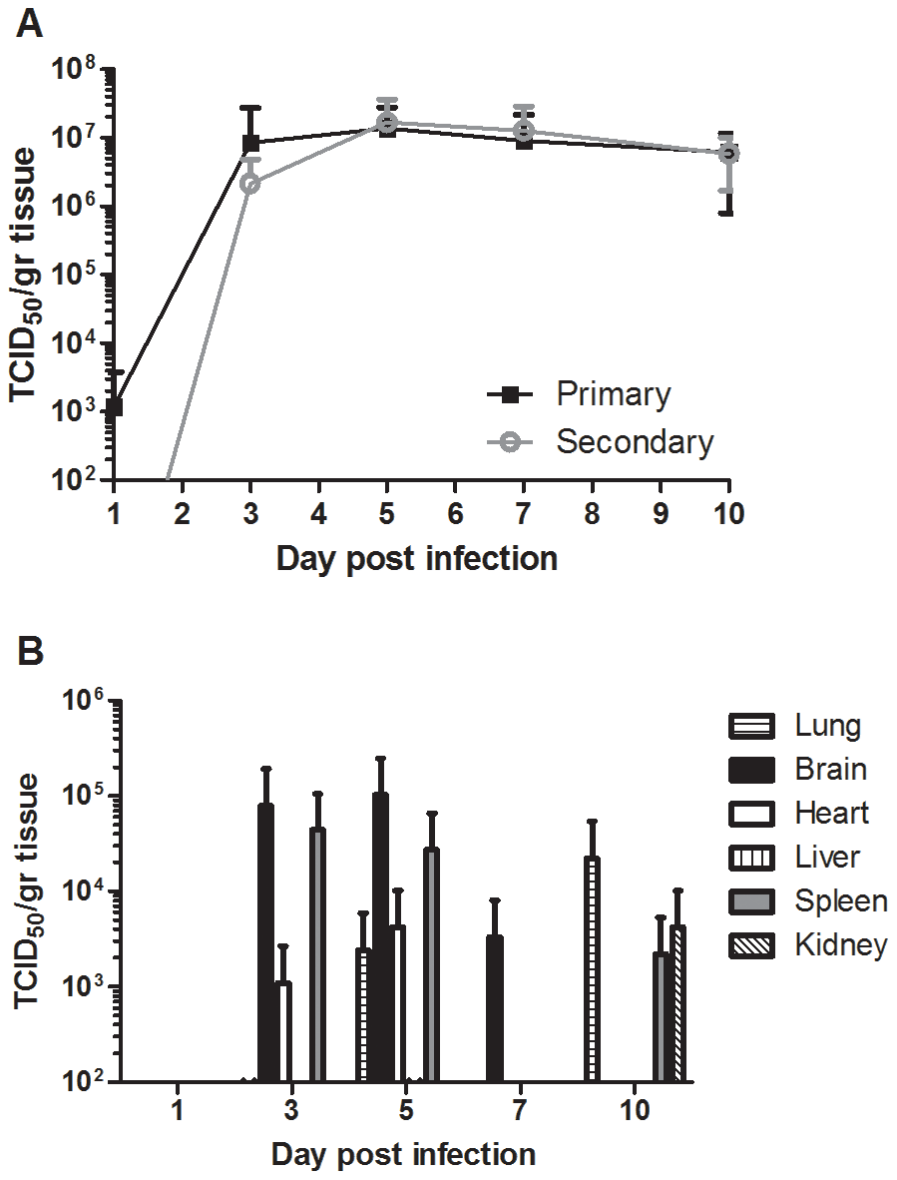
Nipah virus replication in human lung xenografts. Virus replication was determined in human lung (A) and mouse (B) tissues at days 1, 3, 5, 7 and 10 post infection by virus titration. Titers were determined in human lung following primary (direct injection) or secondary (infection due to viremia). Samples from three animals were assayed and analyzed and the mean titers were calculated as TCID_50_/gram tissue. The error bars represent the standard deviation.

Natural NiV infection involves exposure to the virus through the respiratory epithelium. To mimic this route in human lung xenografts that lack air exchange, tissues were directly injected with NiV. Following direct intragraft injection of the 3 lung tissues on the left side (primary infection) within each mouse, NSG mice did not show any signs of morbidity or mortality during our observation period of 10 days. In addition, two non-grafted NSG mice that were challenged intradermally as controls, with the same dose as xenografts, did not develop any clinical signs. Primary infection of the human lung xenografts resulted in detection of infectious NiV as early as 1 day post infection (Figure 2A) and NiV replicated to high titers (10^7^ TCID_50_/gram tissue) by day 3 post infection. NiV titers remained high until the end of the experiment at 10 days post infection. Importantly, high titers of NiV were also detected in the other 3 lung tissues (on the right side of each mouse) that were not initially infected through direct intragraft injection as early as 3 days post infection. This finding clearly demonstrates that the virus can spread from infected human lung grafts to uninfected grafts in the same mouse, most likely through viremia (secondary infection; Figure 2A). The presence of viremia was further supported by the observation that infectious NiV was detected, albeit at lower levels, in several mouse tissues including lung, brain, heart, spleen and kidney at various time points post infection (Figure 2B). In fact, viremia was detected in a blood sample of 1 animal on day 10 post infection in which a low level (300 TCID_50_/mL) of infectious NiV was determined (Table 1). Interestingly, virus was not detected in organs from non-grafted NSG mice that were challenged intradermally with the same dose (Table 1), suggesting the NSG mouse tissues are probably not intrinsically susceptible to NiV. In order to confirm that the human lung xenografts could be infected via the hematogenous route, we next challenged 2 lung-engrafted NSG mice with NiV via the IP route. The IP challenge with NiV in this model confirmed that infection resulted in detectable viremia in 1 animal with virus spreading to the human lung xenografts in both, replicating to high titers and resulting in histopathological changes similar to those observed with intragraft challenge (Table 1). Together, these data suggest that following intragraft infection, the human lung is highly susceptible to NiV infection and results in viremia and subsequent spread to other organs in the absence of disease.

**Table 1:** Summary of virological and histological findings in human lung graft, mouse tissues and blood following Nipah virus challenge.

Route of infection	Human lung			Mouse tissue			
	*Infectious Virus*	*Viral Antigen*	*Histopathology*	*Infectious Virus*	*Viral Antigen*	*Histopathology*	*Blood*
**Direct (n = 15; Primary)**	+++	+++	+++				
**Indirect (n = 9; Secondary)**	+++	+++	+++	+	+; starting 5DPI in lung	−	+
**IP** * **(n = 2)**	+++	+++	+++	+	+; in lung	−	+
**SC** # **(n = 2)**	N/A	N/A	N/A	−	−	−	−

N/A = Not applicable. Direct = injection into the graft, resulting in primary infection; Indirect = secondary infection resulting from virus dissemination from the direct infected lung graft. IP = Intraperitoneal challenge. SC = Subcutaneous challenge. Infectious virus was determined in human lung and mouse tiisues and whole blood by TCID_50%_ as described in Materials & Methods. Viral antigen was detected with an anti N antibody as described in Materials & Methods. Histopathology is based on H&E stained lung sections described in Materials & Methods.

* = data available on day 10 post infection only.

# = for control NSG mice only.

+++ = high virus titer/intense immunostaining/extensive histopathological changes. + = low virus titer/low intensity immunostaining, − = not detected.

### NiV induced histopathology in human lung xenografts

**Figure 3:**
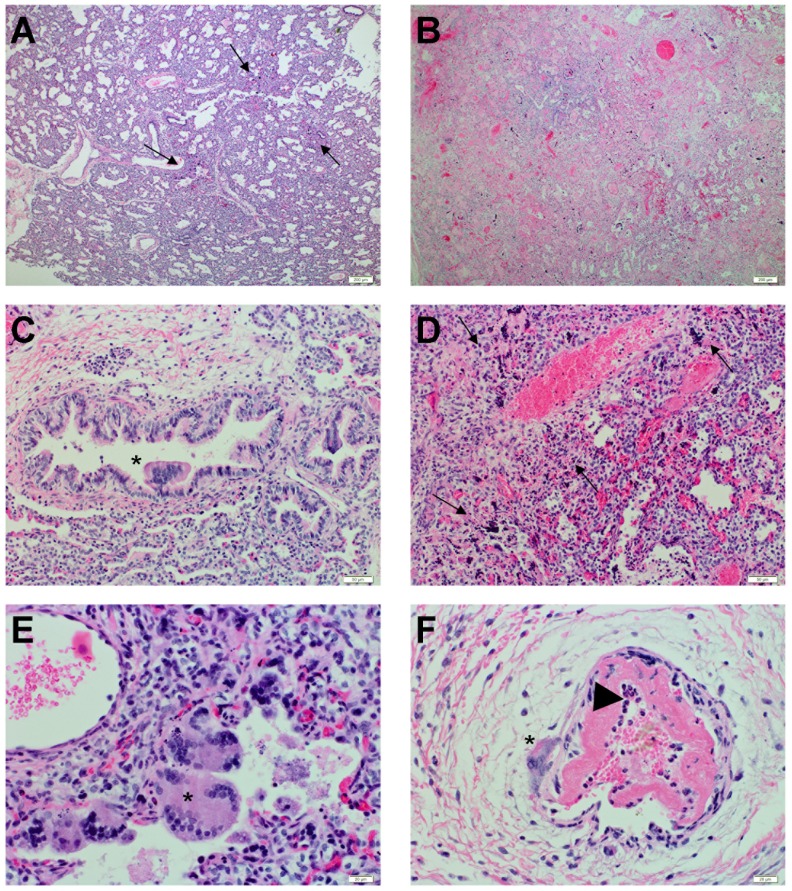
Histopathological changes during Nipah virus infection in human lung xenografts. Human lung sections were stained with H&E as described in Experimental Procedures. (A) Human lung with focal areas of necrosis and syncytia formation (black arrow) on day 3 post infection (10× magnification). (B) Human lung with extensive areas of necrosis, syncytia formation and loss of architecture on day 10 post infection (10× magnification). (C) Bronchi with syncytia formation (*) on day 3 post infection (20× magnification). (D) Loss of alveolar architecture and areas of necrosis (black arrow) on day 10 post infection (20× magnification). (E) Alveolar space with syncytial formation (*) on day 3 post infection (40× magnification). (F) Pulmonary vasculature with syncytial formation (*) on day 3 post infection (40× magnification), fibrinoid necrosis of the intima, and influx of granulocytes (black arrowhead). Data are representative from 6 tissues per animal, 3 animals per time point.

In order to study the histopathological changes associated with NiV infection in the human lung, tissue sections were stained with hematoxylin and eosin (H&E). No gross pathologic lesions were observed in the human lung grafts. Since NSG mice exhibit multiple defects in innate and adaptive immunity [24], NiV infection in human lung grafts did not result in significant inflammation. Histopathological changes in the human lung tissues following NiV infection were independent of the route of infection (intragraft, indirect or intraperitoneal) and included small focal areas with syncytia and necrosis as early as day 3 pi (Figure 3A). These areas rapidly expanded to large areas with hemorrhages and significant loss of architecture of the small airways by day 10 pi (Figure 3B). The main histopathological features of NiV infection in these tissues were the characteristic syncytia formation (Figure 3C) and areas of necrosis (Figure 3D). Syncytia formation could be observed in bronchial epithelium (Figure 3C), alveolar epithelium (Figure 3E) and vascular endothelium (Figure 3F). In addition, fibrinoid necrosis was observed in some of the vasculature as well as recruitment of granulocytes (Figure 3F). In agreement with the absence of clinical signs, NiV infection did not result in histopathological changes in any visceral mouse tissue (Table 1).

### NiV tropism in human lung xenografts

**Figure 4:**
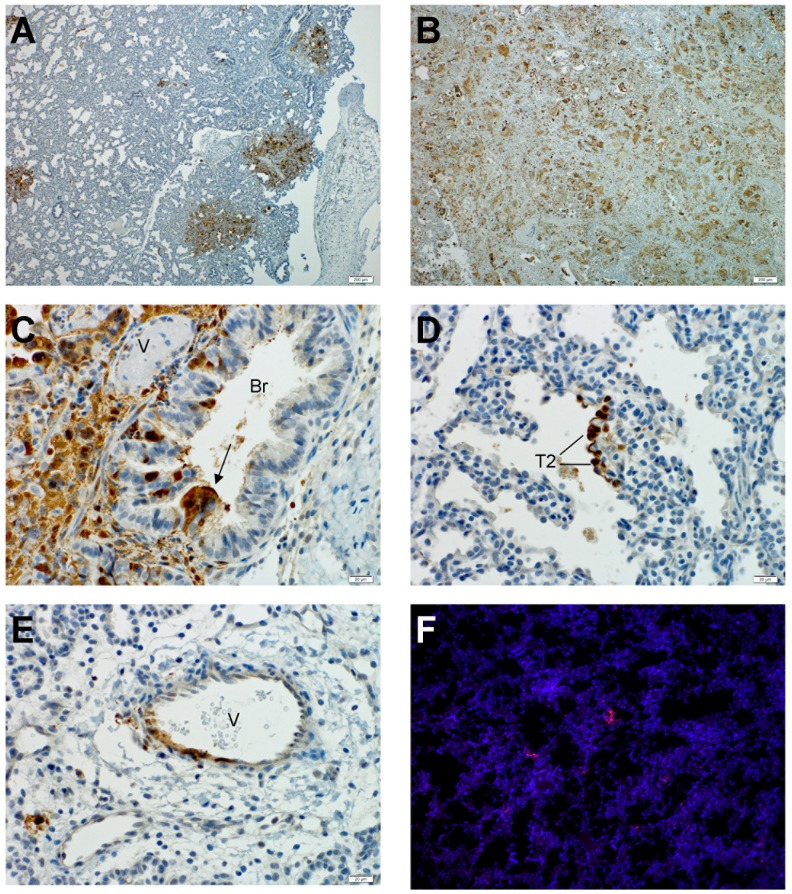
Nipah virus tropism in human lung xenografts. Lung sections were stained by immunohistochemical (IHC) detection of Nipah virus nucleoprotein as described in Experimental Procedures. An overview of viral antigen distribution is shown on day 3 (A) and day 10(B) post infection (10× magnification). (C) Bronchial epithelium positive for NiV antigen in a syncytium (black arrow) on day 3 post infection (40× magnification). (D) Alveolar space with NiV positive cell, primarily cuboidal morphology on day 3 post infection (40× magnification). (E) Pulmonary vasculature positive for NiV antigen on day 3 post infection (40× magnification). (F) Small focal area in mouse lung tissue positive for NiV antigen (red) on day 10 post infection (10× magnification)(nuclei in blue). Br = bronchi, V = pulmonary vasculature. T2 = Type 2 pneumocyte. Data are representative from 3 animals per time point.

In order to identify the cells targeted by NiV in the human lung, viral nucleocapsid protein (N) expression in human lung grafts was examined with immunohistochemistry. Expression of NiV N coincided with the focal areas of histopathological changes on day 3 pi and showed intense staining (Figure 4A). By day 10, widespread expression of NiV N was observed throughout the human lung tissues (Figure 4B). NiV primarily targeted the respiratory epithelium of the bronchi and bronchioles, interstitial mesenchymal cells (Figure 4C), and the small airways (Figure 4D). Cells targeted in the small airways were primarily cuboidal, which is consistent with type-2 pneumocyte morphology, although cells with type-1 pneumocyte morphology also showed reactivity. In addition to the respiratory epithelium, NiV replication also involved the vasculature (Figure 4E). In agreement with the observation that low levels of infectious virus were detectable in several mouse tissues, small focal areas of viral antigen primarily focused in small airway epithelium could be detected in mouse lungs (Figure 4F) but not in other organs tested (Table 1). Tropism of viral antigen in mouse lung was similar between tissues infected by direct injection or following IP challenge (data not shown).

**Figure 5:**
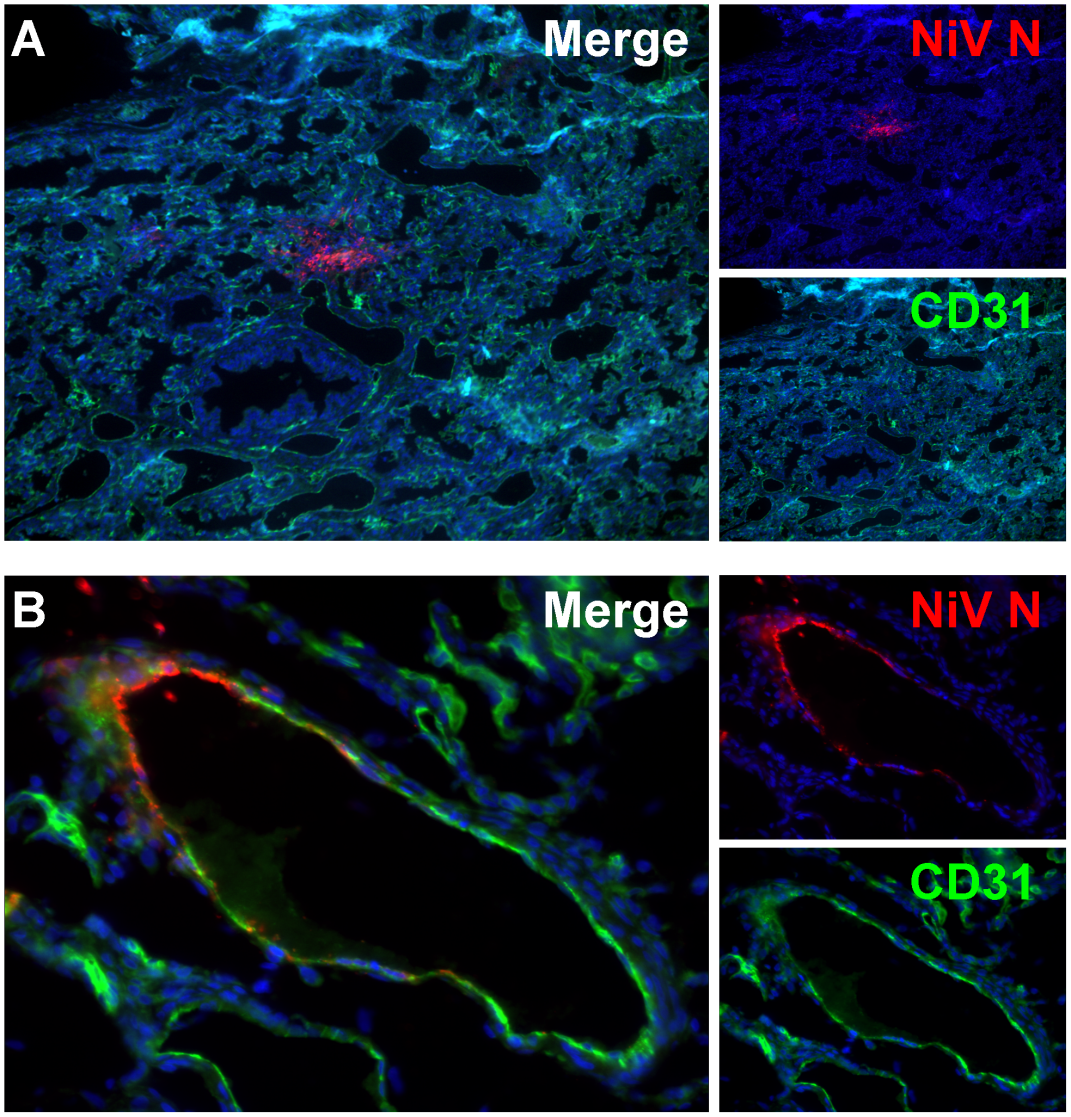
Cell tropism of Nipah virus in human lung xenografts. Lung sections were stained by immunofluorescent detection of Nipah virus nucleoprotein (red), CD31 (green) and nucleus (blue) as described in Experimental Procedures. (A) Human lung showing focal area of Nipah virus distinct from pulmonary vasculature on day 3 post infection (10× magnification). (B) CD31 positive endothelium of pulmonary vasculature is positive for Nipah virus antigen (40× magnification). Data are representative from 3 animals per time point.

Although focal areas were generally not centered around vessels (Figure 5A) during the early stages of infection, when NiV infection involved the vasculature, CD31-positive endothelial cells were a specific target of infection (Figure 5B). Similar findings were observed in animals challenged via the IP route (Table 1).

### Inflammatory responses during NiV infection

**Figure 6:**
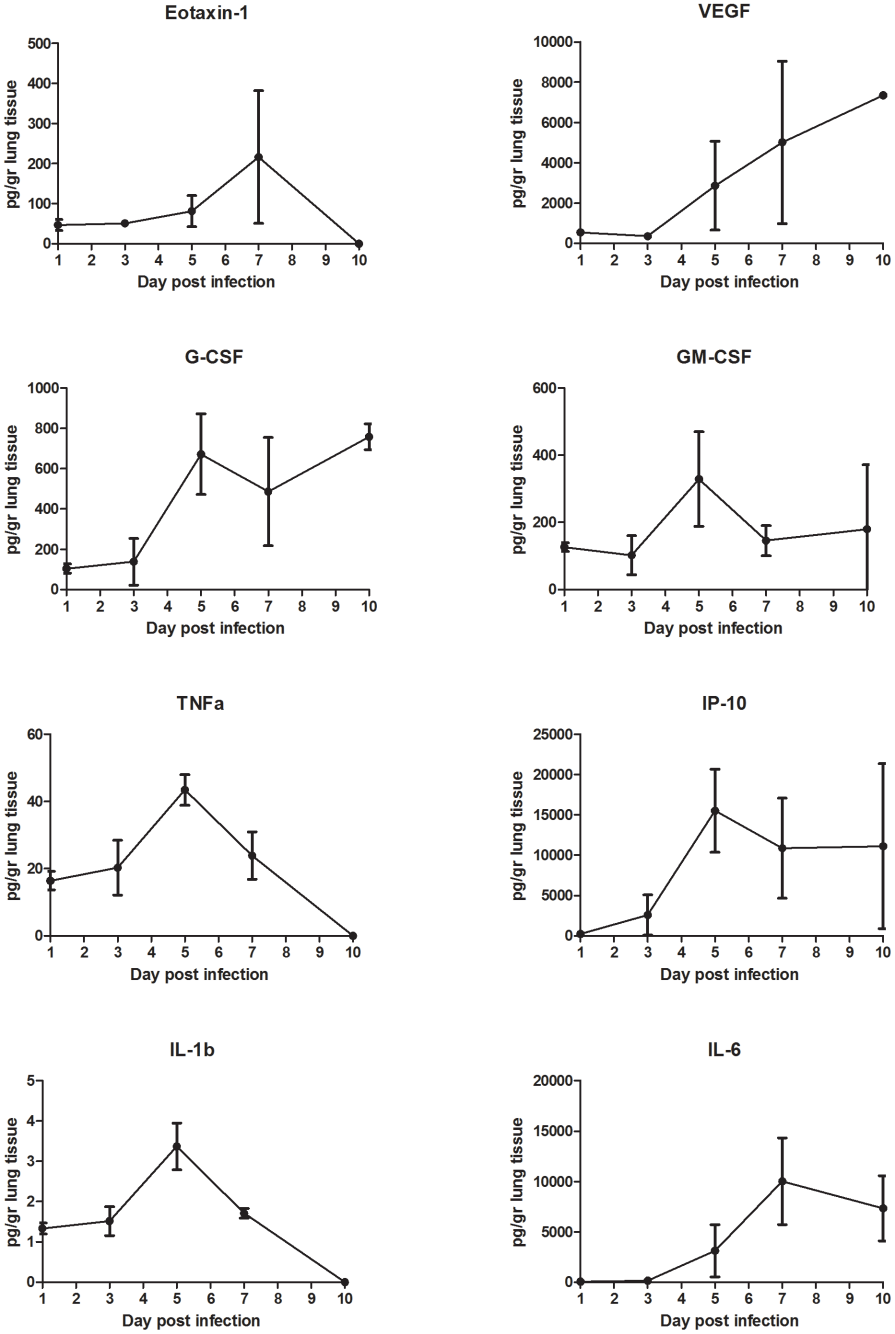
Cytokine levels in Nipah virus infected human lung xenografts. The concentrations of cytokines were determined in lung homogenates of human lung xenografts following direct infection with NiV as described in the Experimental Procedures. Concentrations are expressed as picogram (pg) cytokine per gram lung tissue. The error bars represent the standard deviation.

In order to elucidate the host responses following NiV infection in the human lung, the expression of several cytokines and chemokines was determined in homogenates of human lung xenografts following direct infection with NiV (Figure 6). Since human immune cells were absent in this model, any expression of human cytokines or chemokines was primarily the result from NiV infection of human epithelial and endothelial cells. NiV infection in human lung resulted in the expression of several cytokines/chemokines, including eotaxin-1, G-CSF, GM-CSF, TNFα, VEGF, IP-10, IL-1β and IL-6 starting by day 5 pi (Figure 6). Expression of GM-CSF, TNFα, IP10 and IL-1β peaked on day 5 post infection and gradually declined over time. IL-6 and eotaxin-1 expression peaked at day 7 pi, whereas G-CSF initially peaked on day 5 but remained high throughout infection. Interestingly, VEGF expression continued to increase over time concomitant with the increased hemorrhaging and remodeling of the lung. The cytokine and chemokines profiles were similar between lung xenografts following primary (direct) or secondary (indirect) infection (data not shown).

## Discussion

Nipah virus is an emerging zoonotic virus that can cause severe respiratory distress and encephalitis in humans [1]. Despite intensive studies *in vitro* and in animal models, little is known about the mechanisms governing the development of NiV-related respiratory disease in humans; this is due to difficulties in obtaining human samples where the disease is endemic. To address this important limitation, the goal of the present study was to characterize a novel human lung xenograft model to study the pathogenesis of NiV infection in human lung *in vivo*.

Studies on the molecular mechanisms of NiV-mediated pathogenesis have been hampered by the lack of biologically relevant *in vitro* models for studying the initial host responses to NiV infection in the human lung [7], [13], [15]. To fill this gap, we recently showed that NiV can efficiently replicate in primary epithelial cells from the human respiratory tract [12]. While this is an attractive model to study the early steps of NiV entry in the host, it lacks the complexity of the microenvironment in the lung. In the current model, we show that human fetal lung tissues grafted on an immunocompromised mouse develop into more mature human lung tissues within 3 months after implantation. Transplanted lung tissues rapidly vascularized and developed bronchioles, lined with columnar epithelium, and alveolar-like spaces closely resembling those seen in normal human lung tissue.

The prototype strain of NiV (Malaysia) was used in this study. While the outbreaks in Malaysia and Singapore have primarily been associated with the development of severe febrile encephalitis with a case fatality rate of 38%, respiratory symptoms were observed (40% of lethal cases) [8]. Interestingly, the more recent outbreaks in Bangladesh and India are associated with a higher prevalence of respiratory disease as well as a significantly higher case fatality rate of 67% to 92% [1], [6]. It is currently unknown whether differences in respiratory involvement are due to genetic difference between the Malaysia and Bangladesh strains of NiV or whether confounding factors are involved, however both NiV strains can replicate efficiently and cause respiratory distress in animals [25], [26]. In addition, no histopathological data is available for human cases of the Bangladesh strain of NiV; therefore, the Malaysia strain was used in this study to allow for comparisons of histopathology and viral tropism. In humans, vasculitis and fibrinoid necrosis in the lungs was observed in the majority of fatal cases of NiV infection [8]. Multinucleated giant cells were occasionally observed in alveolar spaces and showed prominent immunostaining for viral antigen, along with alveolar hemorrhage, edema and pneumonia. Bronchial epithelium rarely showed histopathological changes. Nipah viral antigen could also be observed in the vasculature and rarely in bronchiolar epithelium [8]. We believe that the lack of viral antigen in the bronchial epithelium in fatal human cases is most likely due to timing of sampling. We previously showed, using a hamster model, that the bronchial epithelium is initially targeted by NiV early on during infection followed by rapid spread to the interstitium and involvement of pulmonary vessels [13].

In the present model, NiV replicated to high titers following intragraft injection, and virus was found to primarily replicate in respiratory epithelium of the bronchi and small airways. This is consistent with our previous finding that human respiratory epithelium is highly susceptible to NiV infection [12]. In animal models, the lung is the primary target organ of NiV infection following intranasal challenge [13], [14]. In addition to the respiratory epithelium, NiV replication was also found in the endothelium, a type of cell that has been identified as an important target for NiV [11]. The infection of the vascular system is thought to occur in the late stages of disease and lead to systemic spread of these viruses to other organs, including brain and kidney [13], [27]. In our model, systemic spread of the virus was indicated by similar titers and replication kinetics of NiV in directly inoculated lung grafts and grafts not directly injected with virus. This suggests that following infection of the lung, NiV quickly becomes viremic and spreads to other organs. In addition, systemic infection through intraperitoneal injection with NiV also resulted in infection of the human lung grafts, thus confirming hematogenous spread of the virus.

Interestingly, NiV infection in NSG mice engrafted with human lung tissues did not result in clinical signs despite evidence of replication in mouse organs, including lung and brain. Previous studies have shown that NiV infection in type I IFN receptor knock-out mice and aged mice is lethal [28], [29]. Aged mice have been shown to mount an aberrant IFN response [30]. While the NSG mice are immunocompromised, they can mount an IFN response, which seems sufficient to protect against lethal disease in our studies. Alternatively, it is possible that NSG mice are not susceptible to NiV and that the virus measured in mouse organs is only found in the blood. Also, the small foci of viral antigen detected in mouse lungs could correspond to emboli of infected human cells that slough off from the infected grafts.

Several cytokines and especially IL-6, IP-10 and VEGF were upregulated during NiV infection in the human lung. Interestingly, the levels of cytokines observed in our xenograft lung model are similar to those observed in the lungs of fatal cases of influenza virus A (H1N1) [31]. Upregulation of inflammatory mediators such as TNF-α, IP-10, IL-1β and IL-6 in the lungs was previously shown to play a role in the pathogenesis of lethal NiV in hamsters [13], as well as in the development of ALI with other respiratory virus infections, including SARS-CoV and influenza virus (H5N1) [30], [32], [33]. VEGF has an important role in ALI pathogenesis by acting as a growth factor and increasing vascular permeability [34]. We previously showed that VEGF is expressed by human respiratory epithelial cells during NiV infection [12]. This suggests that VEGF may be partly responsible for the increased pulmonary hemorrhage, endothelial destruction, and alveolar remodeling in an emphysema-like phenotype as observed in our model. Since these inflammatory mediators also play an important role in the recruitment of immune cell, our data suggests that inflammation could be observed in this model when human immune cells are present.

In addition to implanting human lung xenografts, the NSG mice have been used to engraft the human hematopoietic system to study hematopoiesis, immunity, inflammatory disease and human-specific pathogens. This humanized NSG mouse model routinely contains >25% human CD45+ cells in the peripheral blood 12 weeks post engraftment of hematopoietic stem cells [35]. Many of the inflammatory mediators expressed in the current study play an important role in immune cell recruitment [36], [37]. The ability to engraft human immune cells will allow us to study the effect of these mediators on specific immune cells populations. Future studies will make use of the fully humanized lung xenograft model to study the role of the inflammatory response in the pathogenesis of the different henipavirus strains in the human lung.

In conclusion, these data confirm that the human lung is highly susceptible to NiV infection. NiV is capable of replicating to high titers in the human lung and targets both respiratory epithelium and endothelium. Infection results in the characteristic syncytial formation and extensive lung damage. Key inflammatory mediators such as IL-6, IP-10, G-CSF and GM-CSF are expressed during infection. This model will allow for more detailed studies of the pathogenesis of respiratory disease caused by henipavirus infection. Furthermore, these data point to several inflammatory mediators that potentially play critical roles in henipavirus pathogenesis, which may be valuable as candidates for future studies of the mechanism of henipavirus pathogenesis and as potential targets for treatment.

## Materials and Methods

### Ethics statement

Approval for animal experiments was obtained from the Institutional Animal Care and Use Committee, University of Texas Medical Branch (protocol number 0905041). Animal work was performed by certified staff in an Association for Assessment and Accreditation of Laboratory Animal Care (AAALAC) approved facility. Animal housing, care and experimental protocols were in accordance with NIH guidelines of the Office of Laboratory Animal Welfare. Discarded tissue from deceased human fetuses was obtained via a non-profit partner (Advanced Bioscience Resources, Alameda, CA) as approved under exemption 4 in the HHS regulations (45 CFR Part 46). Need for informed consent was waived by the UTMB Institutional Review Board.

### Viruses and cells

NiV (Malaysia strain) was kindly provided by the Special Pathogens Branch of the Centers for Disease Control and Prevention, Atlanta, Georgia, United States. The virus were propagated on Vero cells in Dulbecco's minimal essential medium supplemented with 10% fetal calf serum (Hyclone, Logan, UT), L-glutamine, penicillin and streptomycin at 37°C in a humidified CO2 incubator (5%). All infectious work was performed in a class II biological safety cabinet in a biosafety level 4 laboratory (BSL4) at the Galveston National Laboratory.

### Human lung xenograft mouse model

NOD.Cg-Prkdcscid Il2rgtm1Wjl/SzJ mice, also known as NOD/SCID/γcnull or NSG mice (Jackson Laboratories), 3–5 weeks of age, were housed in a sterile microisolator environment. Mice were engrafted with human lung tissue (Advanced Bioscience Resources, Alameda, CA). Six fragments of human fetal lung from the same donor (15–19 weeks of age) were sutured to muscle fascia in the dorsal subcutaneous space in each mouse (∼0.5 cm from the spine, three on each side). Animals received appropriate post-surgery treatment including antibiotics and analgesics.

### Animal infection

Twelve weeks post-engraftment, animals were transferred to an ABSL-4 facility. Prior to infection, animals were anesthetized by chamber induction (5 Liters/min 100% O2 and 3–5% isoflurane). Three of the six lung tissues were inoculated via intragraft injection of 10^5^ TCID_50_ NiV in a 50 µl volume. Animals were monitored daily for weight loss and clinical signs. Groups of 3 animals were euthanized on days 1, 3, 5, 7 and 10 post infection, and samples for virus isolation and histological examination were procured from whole blood (EDTA vacutainer), human lung tissues, and mouse liver, spleen, kidney, lung, heart and brain. In a separate experiment, 2 animals were injected via the intraperitoneal route with 10^5^ TCID_50_ NiV and euthanized 10 days post infection. Control groups were NSG mice without a lung xenograft and challenged via the intradermal route with 10^5^ TCID_50_ NiV on the back of the mouse at the same location the human lung xenografts would be.

### Virus titrations

Whole blood was tested for presence of infectious virus by 10-fold diltutions as described below. Tissue samples were weighed and homogenized in 10 equivalent volumes of DMEM to generate a 10% solution. The solution was centrifuged at 10,000 rpm under aerosol containment in a table top centrifuge for 5 min to pellet insoluble parts. Virus titration was performed using a TCID50 assay on 96-well plates (1×10^4^ Vero cells per well) with 100 µL inocula (cleared homogenate or whole blood) from 10-fold serial dilutions. Plates were incubated for 3 days at 37°C, and wells were scored for cytopathic effect (CPE). Virus concentrations were calculated as TCID_50_ per gram of tissue.

### Histopathology and immunohistochemistry

All tissue samples were immersion-fixed in 10% neutral buffered formalin for at least 7 days under BSL4 conditions. Prior to removal from the BSL4 laboratory, formalin was changed and specimens were processed under BSL2 conditions by conventional methods, either embedded in paraffin, sectioned at 5 µm thickness and stained with hematoxylin and eosin (H&E) or embedded in Tissue Tek and frozen sections cut at 3–8 µm thickness and used for immunofluorescent (IF) staining. Tissues for immunohistochemistry (IHC) were stained as previously described using a rabbit anti-NiV-nucleoprotein (N) antibody (kindly provided by Dr. C. Broder, Uniformed Services University, Bethesda, MD) [13]. Tissues for IF were stained with a rabbit anti-NiV-N antibody, a biotinylated anti-CD31 (eBioscience), anti-collagen IV labeled with Alexa 647 (eBioscience), anti ephrin B2 (Santa Cruz Biotechnology) or ephrin B3 (R&D Systems) and Hoechst for nuclear staining. NiV N in mouse tissue could only be detected following immunofluorescent staining, likely due to the limit of detection by conventional IHC. An Alexa 546 labeled secondary antibody (Life Technologies) was used for detection of the anti NiV N antibody as well as anti ephrin B2 and B3 antibodies and an Alexa 488 conjugated streptavidin (R&D Systems) was used for detection of the anti-CD31 antibody.

### Milliplex analysis

Cytokine/chemokine concentrations in the homogenates of NiV infected human lung tissues were determined using a Milliplex Human Cytokine PREMIXED 28 Plex Immunoassay Kit (Millipore, Billerica, USA). Prior analysis, samples were inactivated on dry ice by gamma-radiation (5 MRad). The assay was performed according to the manufacturer's instructions. The concentration of the following 28 cytokines were determined using the Bio-Plex 200 system (BioRad): Epidermal Growth Factor (EGF), Granulocyte-Colony Stimulating Factor (G-CSF), Granulocyte Macrophage-Colony Stimulating Factor (GM-CSF), interferon (IFN)-α2, IFNγ, Interleukin (IL)-1α, IL-1ß, IL-2, IL-3, IL-4, IL-5, IL-6, IL-7, IL-8, IL-10, IL-12(p40), IL-12(p70), IL-13, IL-15, IL-17A, chemokine ligand 3-like 1 (CCL3L1 or MIP-1α), chemokine ligand 4 (CCL4 or MIP-1ß), chemokine ligand 10 (IP-10 or CXCL10), chemokine ligand 11 (CCL11 or Eotaxin-1), chemokine ligand 13 (CCL13 or MCP-1), Tumor Necrosis Factor (TNF-α), Lymphotoxin alpha (TNFß) and Vascular Endothelial Growth Factor A (VEGF).
